# Association of thyroid autoimmunity with extra-thyroid diseases and the risk of mortality among adults: evidence from the NHANES

**DOI:** 10.3389/fendo.2024.1323994

**Published:** 2024-02-09

**Authors:** Jun-Long Song, Jia-Wei Hu, Ling-Rui Li, Zhi-Liang Xu, Juan-Juan Li, Sheng-Rong Sun, Chuang Chen

**Affiliations:** Department of Breast and Thyroid Surgery, Renmin Hospital of Wuhan University, Wuhan, Hubei, China

**Keywords:** thyroid autoimmunity, TgAb, TPOAb, extra-thyroid disease, mortality, NHANES

## Abstract

**Background:**

Thyroid autoimmunity is one of the most prevalent autoimmune diseases. However, its association with extra-thyroid diseases and mortality risk in the general population remains uncertain. Our study aims to evaluate the association of thyroid autoimmunity with extra-thyroid disease and the risk of mortality.

**Methods:**

A prospective cohort study was conducted using data from the National Health and Nutrition Examination Survey (NHANES) with participants from 2007–2008, 2009–2010, and 2011–2012, tracking their mortality until 2019. Associations between thyroid autoimmunity, which was defined as having positive thyroid peroxidase antibody (TPOAb) and/or thyroglobulin antibody (TgAb), and extra-thyroid disease including diabetes, hypertension, cardiovascular disease, chronic lung disease, arthritis, cancer and chronic renal disease and the risk of mortality were investigated.

**Results:**

A total of 7431 participants were included in this study. Positive The prevalence of positive TgAb was 7.54%, and positive TPOAb prevalence was 11.48%. TgAb was significantly associated with diabetes (Model 1: OR=1.64, 95% CI:1.08-2.50; Model 2: OR=1.93, 95% CI: 1.21-3.08) and hypertension (Model 1: OR=0.67, 95% CI: 0.49-0.91; Model 2: OR=0.62, 95% CI: 0.44-0.88). TPOAb was associated with a lower prevalence of chronic lung disease (model 1: OR=0.71, 95% CI: 0.54-0.95; model 2: OR=0.71, 95% CI: 0.53-0.95). No associations were observed between TgAb, TPOAb and other extra-thyroid diseases. Neither TgAb nor TPOAb were associated with all-cause mortality or heart disease mortality.

**Conclusion:**

TgAb was linked to a higher prevalence of diabetes and a lower prevalence of hypertension, while TPOAb was associated with a decreased prevalence of chronic lung disease. However, neither TgAb nor TPOAb posed a risk for all-cause mortality or heart disease mortality.

## Introduction

Thyroid autoimmunity results from aberrations in the immune system, leading to an autoimmune attack on the thyroid gland (1). It accounts for 30% of all autoimmune diseases, making it one of the most prevalent types (2). Serum thyroid autoantibodies, particularly thyroid peroxidase antibody (TPOAb) and thyroglobulin antibody (TgAb), are hallmarks of thyroid autoimmunity. Underlying thyroid autoimmunity without clinical autoimmune diseases is diagnosed by the presence of these antibodies. Thyroid autoimmunity encompasses a closely related spectrum of disorders that mainly include Hashimoto’s thyroiditis (HT) and Graves’ disease (GD). HT, also called chronic lymphocytic or autoimmune thyroiditis, is the most frequent thyroid autoimmune disorder and was first described in 1912 by Hakaru Hashimoto (3). It is characterized by increased thyroid volume, lymphocyte infiltration of the parenchyma, and the presence of antibodies specific to thyroid antigens. In the case of HT, thyroid autoimmunity leads to follicular cell damage and the development of hypothyroidism (4). In contrast, GD, which is mainly associated with hyperthyroidism, is characterized by a primarily humoral response and the presence of anti-thyroid stimulating hormone (TSH) receptor antibodies (1, 4).

The etiology and mechanism of thyroid autoimmunity remain elusive but are considered multifactorial, involving susceptibility genes and environmental exposures, including immune system defects, drugs, infections, micronutrients, and molecular mimicry between microbial and host antigens (5, 6). Genetic predisposition, linked to immune-regulatory gene polymorphisms, may disrupt immune tolerance and trigger autoimmunity (7–10). Micronutrients including iodine, iron, and selenium may also play important roles in the pathogenesis of thyroid autoimmunity (11). It is reported that an excess of iodine intake, selenium deficiency, and iron deficiency induce thyroid autoimmunity (12–14).

Thyroid disorders could potentially impact extra-thyroid diseases such as cardiovascular disease or cancer (15–19). Genetic and environmental factors contributing to thyroid autoimmunity might also influence extra-thyroid disease risk and survival. While thyroid autoimmunity is a risk factor for thyroid dysfunction, many individuals with thyroid autoimmunity do not manifest thyroid dysfunction. Previous studies have primarily focused on the association between thyroid dysfunction and the risk of extra-thyroid diseases or mortality. The question of whether thyroid autoimmunity, per se, is linked to an elevated risk of extra-thyroid diseases and mortality in the general population remains unanswered. Some studies have indicated positive associations between thyroid autoimmunity and diabetes (20–22) as well as an increased risk of breast cancer (23–25). However, these studies often have small sample sizes and retrospective designs, with few investigating the impact on survival. Thus, research examining the association of thyroid autoimmunity with the risk of extra-thyroid diseases and mortality is warranted. This population-based study sought to evaluate these associations using data from the National Health and Nutrition Examination Survey (NHANES).

## Methods

### Study population

NHANES is a nationally representative dataset comprising a multistage, stratified, and clustered probability sample of noninstitutionalized individuals randomly selected from the U.S. general population. The data include structured interview data, physical examination results, and laboratory testing results, including urine and blood samples. The data are collected in two stages. First, health interviews are conducted in participants’ homes. Then, health measurements and laboratory tests are undertaken in mobile examination centers (MEC). Raw survey data are weighted to population estimates by the National Center for Health Statistics to ensure nationally representative data. Participants were prospectively followed for mortality through December 2019. The NHANES data, along with documents on the survey methods and other information, are publicly available on the NHANES website (https://www.cdc.gov/nchs/nhanes/about_nhanes.htm). The NHANES study protocols were approved by the National Center for Health Statistics (NCHS) institutional review board, and all participants provided informed written consent at enrollment (https://www.cdc.gov/nchs/nhanes/irba98.htm). Because the data were completely deidentified, the institutional review board exempted this analysis from human study considerations.

**Figure 1:**
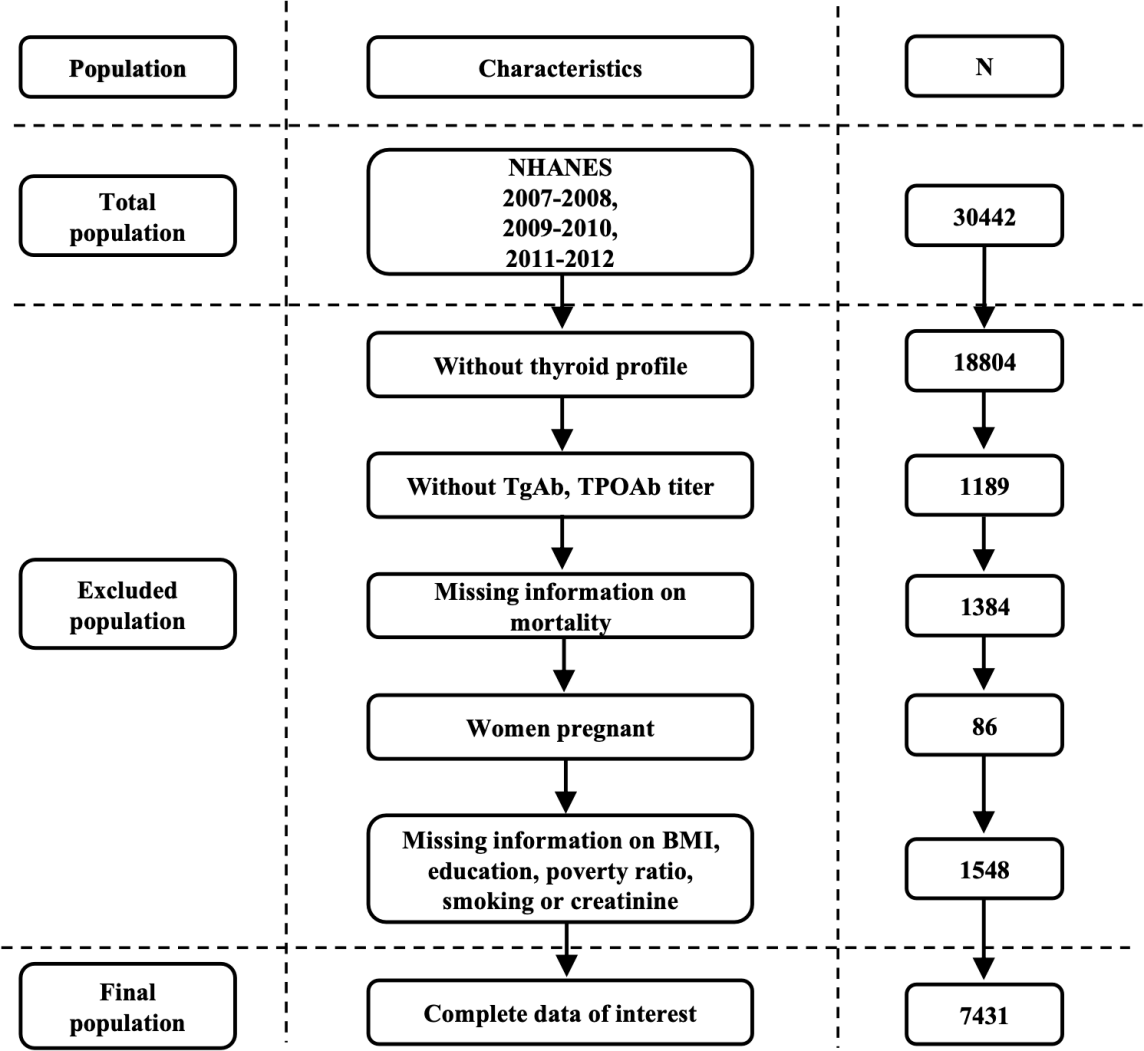
Flow chart of the included participants in the NHANES cohort.

Thyroid laboratory profiles, including for TgAb and TPOAb, were collected from NHANES 2007–2008, 2009–2010, and 2011–2012 participants. **Figure 1** illustrates the study population selection process. A total of 11,638 participants were enrolled initially, but those missing data on TgAb or TPOAb titers (n=1,189), mortality information (n=1,384), pregnant or lactating at the time of blood draw (n=86), and individuals with missing data on body mass index (BMI), education, poverty ratio, smoking, or creatinine (n=1,548) were excluded. The final analytical cohort consisted of 7,431 participants.

### Definition of thyroid autoimmunity

TgAb and TPOAb titers were measured using a sequential two-step immunoenzymatic “sandwich” assay. The manufacturers’ reference ranges (reference range: TgAb, <4 IU/ml; TPOAb, <9 IU/ml) were used to denote the status of TgAb and TPOAb. Subjects with a TgAb titer ≥4 IU/mL and/or a TPOAb titer ≥9 IU/mL were considered positive for thyroid autoantibodies. We also carried out an analysis according to the different statuses of TgAb and TPOAb.

### Other measurements

We included a wide array of demographic data, anthropometric assessments, and comprehensive laboratory data in this study. Race was categorized as Mexican American, non-Hispanic white, non-Hispanic black, and others. Education level was categorized as below high school, high school or equivalent, and above high school. Smoking status was categorized as never smoker, current smoker, and ex-smoker. Family poverty income ratio (PIR), which was calculated as the ratio of family income to poverty, was also included in the model (PIR categories: ≤1, 1< to ≤3, >3). BMI was calculated using height and weight. Serum thyroid stimulating hormone (TSH) levels from participants were measured with a microparticle enzyme immunoassay. The normal reference range for TSH was defined as 0.34 to 5.60 mIU/L according to the laboratory procedure manual. Serum creatinine (Scr) measurements were performed according to the laboratory procedure manual for NHANES 2007–2008, 2009–2010, and 2011–2012. Estimated glomerular filtration rate (eGFR; measured in milliliters per minute per 1.73 m^2^) was calculated using the Chronic Kidney Disease (CKD) Epidemiology Collaboration equation (GFR = 141 × min (Scr/κ, 1) ^α^ × max (Scr/κ, 1) ^-1.209^ × 0.993 ^Age^ × 1.018 [if female] × 1.159 [if black]; κ = 0.7 for females and 0.9 for males, α = -0.329 for females and -0.411 for males, min indicates the minimum of Scr/κ or 1, and max indicates the maximum of Scr/κ or 1) (26).

### Outcome ascertainment

The extra-thyroid diseases in this study include diabetes, hypertension, cardiovascular disease, lung disease, arthritis, cancer and CKD stage 3-5. All of the diseases except CKD were defined using a standardized questionnaire. All participants were asked the following questions: “Has a doctor or other health professional ever told you that you have diabetes/high blood pressure/congestive heart failure/coronary heart disease/angina pectoris/heart attack/stroke/asthma/emphysema/chronic bronchitis/arthritis/cancer?” If an individual had one condition of congestive heart failure, coronary heart disease, angina pectoris, heart attack or stroke, he/she would be considered as having cardiovascular disease. If an individual had one condition of asthma, emphysema or chronic bronchitis, he/she would be considered as having lung disease. CKD stage 3-5 was defined as the eGFR <60 ml/min_*_1.73m^2^.

The mortality of a participant in the NHANES was ascertained by a probabilistic record match to death certificate records from the National Death Index (NDI). The matching method is elucidated on the website of the NCHS (https://www.cdc.gov/nchs/data-linkage/mortality-public.htm). The cause of death was determined according to the International Classification of Diseases, 10th version (ICD–10). The outcomes of interest in this study were mortality from all causes, cancer (codes C00–C97), heart diseases (codes I00–I09, I11, I13, and I20–I51), and other causes.

### Statistical analysis

To interpret the complex NHANES survey design, appropriate sampling weights were used to reconstitute the data on a representative population level for the entire United States. The mean and standard deviation were calculated for the continuous variables and the proportions were calculated for the categorical variables in each category sub-stratified by thyroid autoantibody status. The statistical significance of the differences between groups was evaluated using chi-square tests for the categorical variables and analysis of variance for the continuous variables. Both unadjusted and adjusted associations between thyroid autoantibodies and extra-thyroid diseases were estimated using Logistic regression. Two separate multivariate models were constructed for each disease. Model 1 adjusted for age (continuous), sex (male or female), race/ethnicity (Mexican American, non-Hispanic white, non-Hispanic black, or other race/ethnicity), education status (less than high school, high school or equivalent, or above high school), smoking (never, former, or current), PIR (≤1, 1< to ≤3, or >3), and BMI (<18.5,18.5≤to<25,≥25) and TSH (<0.34, 0.34≤ to ≤5.60, or >5.60). Model 2 further adjusted for further adjusted for previous hypertension (yes, no, or missing), diabetes (yes, no, or missing), cardiovascular disease (yes, no), lung disease (yes, no), cancer (yes, no, or missing), arthritis (yes, no) and CKD stage 3-5. We further performed stratum-specific analyses within subgroups defined according to sex (male or female), age (<50 or ≥50), and euthyroid. We used model 2 to perform subgroup analysis. The Cox proportional hazards model was used to detect the association between thyroid autoantibodies and the risk of mortality. Unadjusted hazard ratios (HR) for the association between thyroid autoantibodies and mortality were estimated using unadjusted Cox proportional hazards models. Multivariable Cox proportional hazards models were adjusted for age, sex, race/ethnicity, education status, smoking, PIR, BMI, TSH, previous hypertension, diabetes, cardiovascular disease, lung disease, cancer, arthritis, and CKD stage 3-5. All analyses were conducted using STATA version 17.0 (STATA Corp, College Station, TX) and R version 4.1.0. All statistical tests were two-tailed, and a p-value <0.05 was considered statistically significant.

## Results

### Characteristics of participants

A total of 7431 participants were included in this study (mean age: 49.80 years; males: 49.09%). Among the included participants, the prevalence of positive TgAb was 7.54% and positive TPOAb prevalence was 11.48%. A total of 1045 individuals (14.98%) had elevated thyroid autoantibodies. The demographics were analyzed for each group and then compared. The results are shown in **Table 1**. Compared to individuals without thyroid autoimmunity, those with thyroid autoimmunity were more likely to be older, female, non-Hispanic white, with more education, and PIR≥3. Furthermore, thyroid autoimmunity was associated with higher TSH levels and lower eGFR. Current smokers had a significantly lower prevalence of thyroid autoimmunity than never smokers and former smokers.

**Table 1:** Baseline characteristics of participants according to TGAb, TPOAb status in the National Health and Nutrition Examination Survey, 2007 to 2012.

Characteristics	TGAb (+)			TPOAb (+)		
	No	Yes	p	No	Yes	p
**Participants, No.**	6906 (92.46)	525 (7.54)	/	6625 (88.52)	806 (11.48)	/
**Age, mean (SD)**	46.42 (0.50)	52.80 (1.03)	<0.01	46.31 (0.50)	51.46 (0.79)	<0.01
Gender						
Male	3526 (50.11)	204 (36.61)	<0.01	3450 (50.92)	280 (34.98)	<0.01
Female	3380 (49.89)	321 (63.39)		3175 (49.08)	526 (65.02)	
Race						
Mexican American	1062 (7.77)	80 (5.13)	<0.01	1013 (7.85)	129 (5.46)	<0.01
Non-Hispanic White	3253 (69.77)	294 (77.41)		3099 (69.06)	448 (80.19)	
Non-Hispanic Black	1412 (10.76)	53 (4.32)		1375 (11.00)	90 (4.63)	
Other	1179 (11.70)	98 (13.14)		1138 (12.08)	139 (9.72)	
Education status						
Under high school	1937 (17.76)	143 (14.56)	0.12	1882 (18.17)	198 (12.45)	0.01
High school or equivalent	1615 (22.69)	103 (19.58)		1529 (22.39)	189 (22.96)	
Above high school	3354 (59.56)	279 (65.86)		3214 (59.44)	419 (64.60)	
Poverty rartio						
<=1	1498 (15.07)	93 (12.22)	0.13	1449 (15.29)	142 (11.48)	0.01
1<to<=3	2965 (36.72)	230 (32.12)		2850 (36.88)	345 (32.52)	
>3	2443 (48.21)	202 (55.66)		2326 (47.83)	319 (56.00)	
Smoking						
Never	3636 (53.71)	293 (62.89)	<0.01	3481 (53.87)	448 (58.50)	<0.01
Ever	1747 (24.67)	147 (24.68)		1665 (24.28)	229 (27.64)	
Current	1523 (21.62)	85 (12.43)		1479 (21.84)	129 (13.86)	
**BMI, mean (SD)**	28.68 (0.15)	28.62 (0.47)	0.90	28.68 (0.16)	28.60 (0.43)	0.84
18.5≤BMI<25	118 (1.64)	8 (2.14)	0.87	111 (1.56)	15 (2.58)	0.49
BMI<18.5	1915 (30.04)	156 (30.78)		1851 (30.11)	220 (29.91)	
BMI≥25	4873 (68.32)	361 (67.09)		4663 (68.32)	571 (67.51)	
Diabetes						
**No**	6096 (91.48)	452 (86.06)	0.01	5851 (91.32)	697 (89.17)	0.26
Yes	806 (8.48)	73 (13.94)		770 (8.64)	109 (10.83)	
Missing or unknown	4 (0.04)	0 (0)		4 (0.04)	0 (0)	
Hypertension						
**No**	4467 (69.19)	332 (69.54)	0.83	4306 (69.52)	493 (66.89)	0.40
Yes	2432 (30.75)	193 (30.46)		2313 (30.43)	312 (33.06)	
Missing or unknown	7 (0.06)	0 (0)		6 (0.06)	1 (0.05)	
Cardiovascular disease						
**No**	6185 (92.13)	459 (89.47)	0.20	5938 (92.26)	706 (89.33)	0.12
Yes	721 (7.87)	66 (8.60)		541 (7.74)	72 (10.67)	
Arthritis						
**No**	4969 (76.11)	347 (69.73)	0.04	4779 (76.20)	537 (71.20)	0.06
Yes	1925 (23.70)	176 (29.99)		1836 (23.65)	265 (28.22)	
Missing or unknown	12 (0.19)	2 (0.29)		10 (0.15)	4 (0.58)	
Chronic lung disease						
**No**	5670 (82.09)	436 (83.32)	0.69	5441 (81.75)	665 (85.50)	0.06
Yes	1236 (17.91)	89 (16.68)		1184 (18.25)	141 (14.50)	
Cancer						
**No**	6252 (90.63)	460 (89.50)	0.15	5995 (90.54)	717 (90.63)	0.04
Yes	648 (9.30)	63 (10.13)		625 (9.42)	86 (8.96)	
Missing or unknown	6 (0.06)	2 (0.37)		5 (0.04)	3 (0.40)	
TSH						
≤0.34	142 (1.67)	24 (4.45)	<0.01	128 (1.54)	38 (4.49)	<0.01
0.34<to<5.6	6642 (96.70)	448 (85.39)		6402 (97.23)	688 (85.25)	
≥5.6	122 (1.63)	53 (10.16)		95 (1.23)	80 (10.26)	
eGFR, mean (SD)	95.17 (0.62)	88.08 (1.24)	<0.01	95.27 (0.60)	89.74 (0.87)	<0.01
≥60	6319 (94.23)	461(90.95)	0.01	6063 (94.20)	717 (92.32)	0.10
<60	587 (5.77)	64 (9.05)		562 (5.80)	89 (7.68)	

SD, standard deviation; BMI, body mass index; TSH, thyroid stimulating hormone; eGFR, estimated glomerular filtration rate.

### Thyroid autoantibodies and extra-thyroid disease.

**Figure 2:**
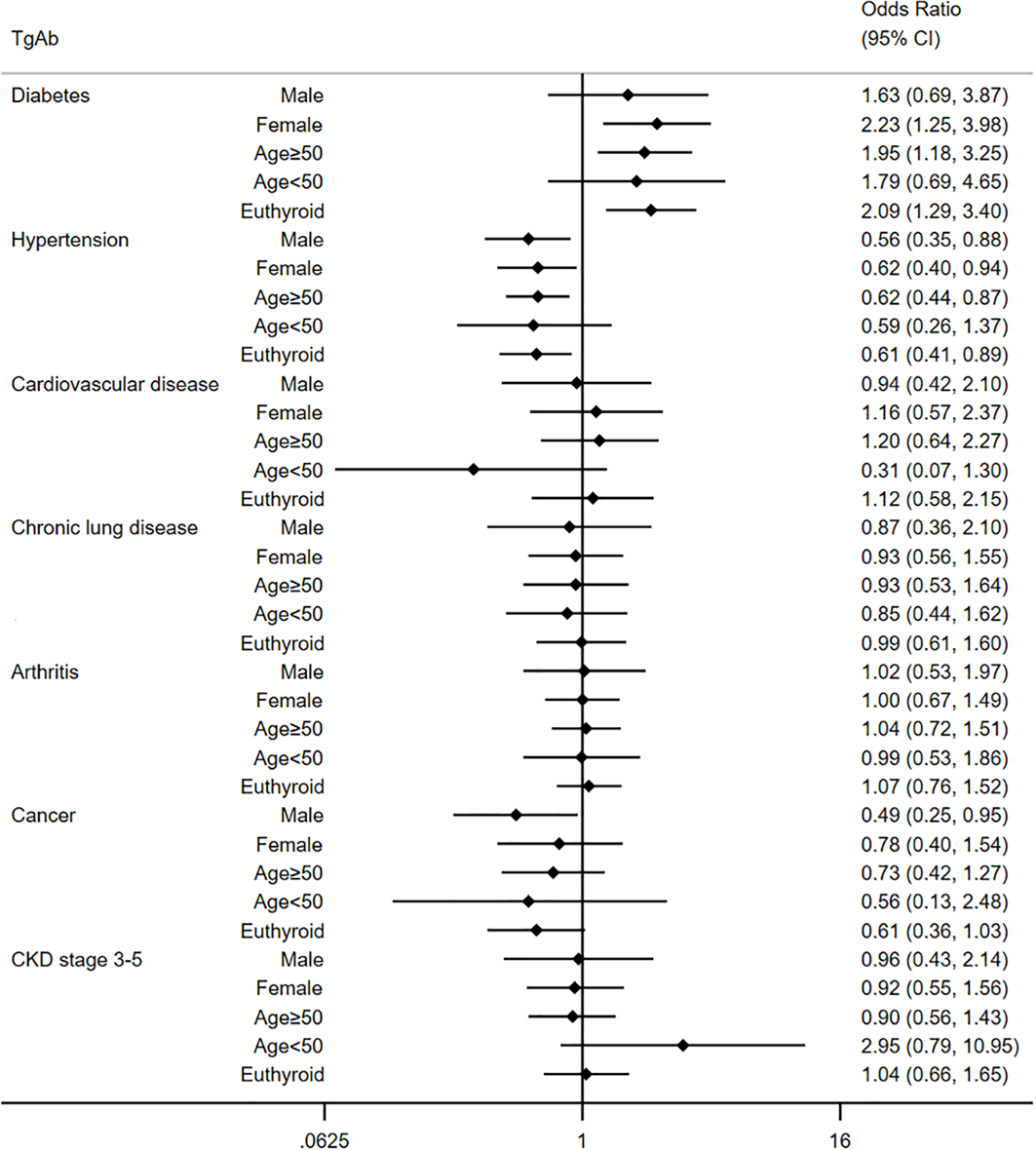
Multivariate analysis for the relationship between TgAb and extra-thyroid disease in different subgroups.

The association between thyroid autoimmunity and extra-thyroid diseases is presented in **Table 2**. In univariate analysis, TgAb was positively associated with diabetes (OR=1.75, 95% confidence interval [CI]: 1.19-2.55), arthritis (OR=1.28, 95% CI: 1.03-1.85), and CKD stage 3-5 (OR=1.63, 95% CI: 1.10-2.40). We performed multivariable analyses to further understand the association. After adjusting confounders in Model 1 and Model 2, TgAb was still significantly associated with diabetes (Model 1: OR=1.64, 95% CI:1.08-2.50; Model 2: OR=1.93, 95% CI: 1.21-3.08). However, TgAb had no obvious association with arthritis and CKD stage 3-5 in both multivariate models. Specially, although no significant association was detected between TgAb and hypertension in univariate analysis (OR=0.99, 95%CI: 0.76-1.28). In multivariate analyses, we demonstrated an inverse association between TgAb and hypertension (Model 1: OR=0.67, 95% CI: 0.49-0.91; Model 2: OR=0.62, 95% CI: 0.44-0.88). As shown in **Figure 2**, we further carried out subgroup analyses considering the potential sex, age and thyroid function differences in TgAb status. A more significant correlation between TgAb and diabetes was observed in females (OR=2.23, 95% CI:1.25-3.98) and individuals over 50 years old (OR=1.95, 95% CI: 1.18-3.25). This correlation appears to be independent of thyroid function. Because TgAb was also significantly associated with diabetes in those individuals with euthyroid (OR=2.09, 95% CI: 1.29-3.40). In the general population, TgAb had no statistically significant association with cancer. However, a significant inverse association between TgAb and cancer was demonstrated in males (OR=0.49, 95% CI: 0.25-0.95) in adjusted subgroup analyses. No significant association was observed between TgAb and cardiovascular disease and chronic lung disease. NHANES did not specify whether the diabetes was type 1 diabetes or type 2 diabetes. As most of the type 1 diabetes were diagnosed in puberty and early adulthood (27), we categorized the diabetes into two groups: diabetes diagnosed before 30 and diabetes diagnosed after 30. We demonstrated a significant association between TgAb and diabetes irrespective of age of diagnosis (**Supplementary Table 1**).

**Table 2:** Associations between TGAb, TPOAb status and extra-thyroid disease in the National Health and Nutrition Examination Survey, 2007 to 2012 stratified by gender, followed up through 2015.

	Patients, No. (%)	Univariate model		Multivariate model			
				Model 1		Model 2	
		OR (95% CI)	P	HR (95% CI)	P	HR (95% CI)	P
TGAb							
**Diabetes**	879 (8.89)	1.75 (1.19-2.55)	<0.01	1.64 (1.08-2.50)	0.02	1.93 (1.21-3.08)	<0.01
**Hypertension**	2625 (30.73)	0.99 (0.76-1.28)	0.91	0.67 (0.49-0.91)	0.01	0.62 (0.44-0.88)	0.01
**Cardiovascular disease**	787 (8.07)	1.38 (0.84-2.27)	0.20	1.01 (0.58-1.77)	0.97	1.07 (0.60-1.92)	0.82
**Chronic lung disease**	1325 (17.82)	0.92 (0.60-1.41)	0.69	0.91 (0.58-1.44)	0.69	0.90 (0.57-1.42)	0.63
**Arthritis**	2101 (24.22)	1.38 (1.03-1.85)	0.03	0.99 (0.74-1.34)	0.97	1.05 (0.76-1.45)	0.76
**Cancer**	711 (9.37)	1.10 (0.71-1.71)	0.66	0.69 (0.41-1.16)	0.16	0.69 (0.40-1.18)	0.17
**CKD stage 3-5**	651 (6.01)	1.63 (1.10-2.40)	0.02	0.96 (0.62-1.49)	0.86	0.96 (0.62-1.47)	0.84
TPOAb							
**Diabetes**	879 (8.89)	1.28 (0.90-1.81)	0.17	1.25 (0.86-1.82)	0.24	1.37 (0.91-2.07)	0.13
**Hypertension**	2625 (30.73)	1.13 (0.88-1.45)	0.33	0.86 (0.65-1.14)	0.28	0.84 (0.64-1.11)	0.22
**Cardiovascular disease**	787 (8.07)	1.42 (0.91-2.24)	0.12	1.18 (0.74-1.88)	0.48	1.25 (0.74-2.11)	0.39
**Chronic lung disease**	1325 (17.82)	0.76 (0.57-1.02)	0.07	0.71 (0.54-0.95)	0.02	0.71 (0.53-0.95)	0.02
**Arthritis**	2101 (24.22)	1.28 (0.99-1.66)	0.06	0.98 (0.71-1.35)	0.90	1.04 (0.76-1.44)	0.77
**Cancer**	711 (9.37)	0.95 (0.64-1.40)	0.79	0.66 (0.42-1.03)	0.07	0.66 (0.41-1.06)	0.08
**CKD stage 3-5**	651 (6.01)	1.35 (0.94-1.95)	0.11	0.96 (0.66-1.39)	0.82	0.91 (0.61-1.36)	0.65

Model 1: adjusted for age, sex, race, poverty index, education, smoking, BMI and TSH.

Model 2: adjusted for age, sex, race, poverty index, education, smoking, BMI, diabetes, hypertension, CVD, chronic lung disease, arthritis, cancer, TSH, eGFR.

**Figure 3:**
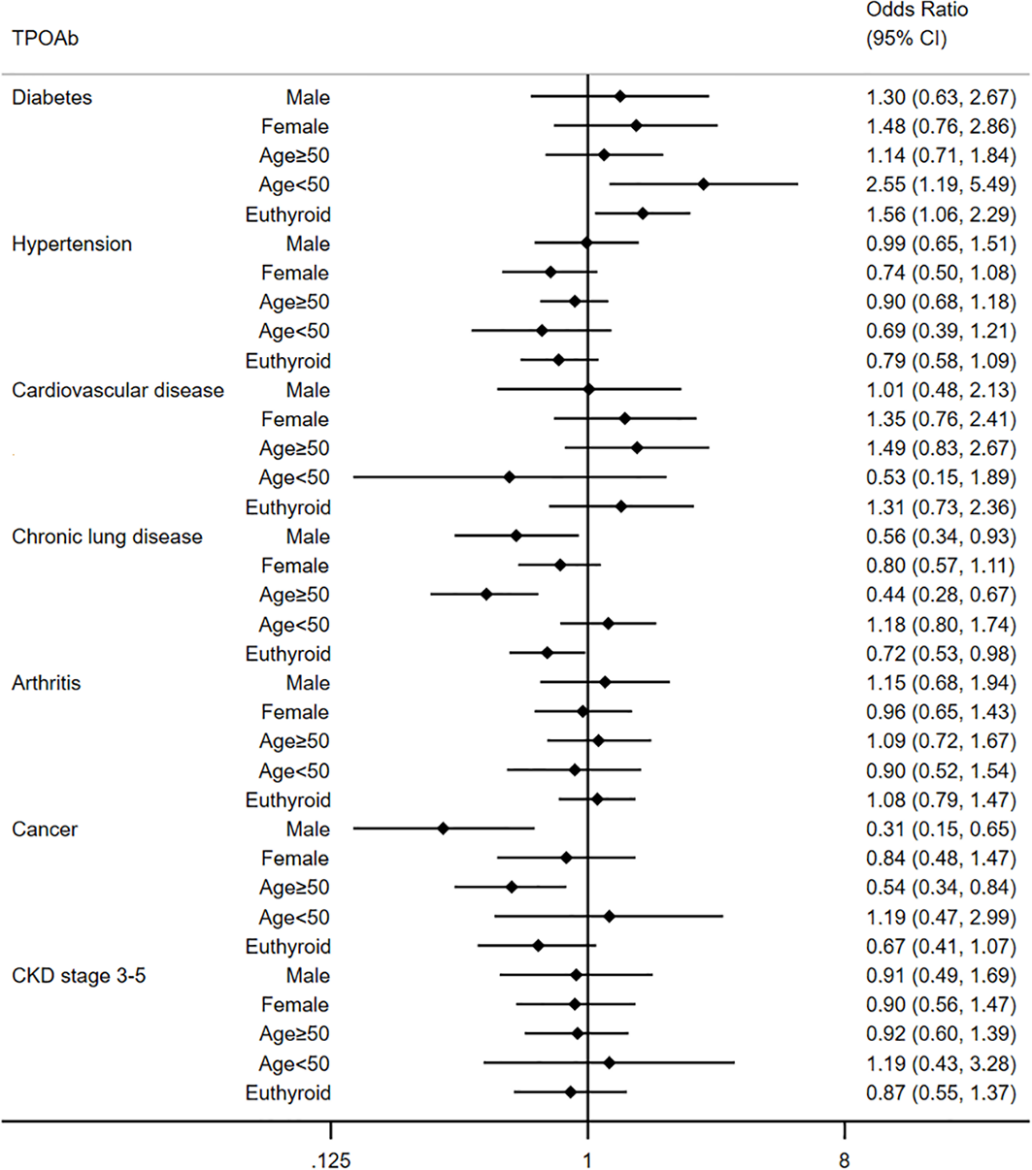
Multivariate analysis for relationship between TPOAb and extra-thyroid disease in different subgroups.

Different from TgAb, no significant association was detected between TPOAb and extra-thyroid diseases in univariate analyses. We only observed a borderline correlation between TPOAb and chronic lung disease (OR=0.76, 95% CI: 0.57-1.02) and arthritis (OR=1.28, 95% CI: 0.99-1.66) in the univariate model. After adjusting for confounders, TPOAb was associated with a lower prevalence of chronic lung disease (Model 1: OR=0.71, 95% CI: 0.54-0.95; Model 2: OR=0.71, 95% CI: 0.53-0.95). We demonstrated no association between TPOAb and other diseases in multivariate analyses. Further subgroup analyses were also carried out to understand the association between TPOAb and extra-thyroid diseases. As shown in **Figure 3**, TPOAb was associated with a higher prevalence of diabetes in individuals less than 50 years old (OR=2.55, 95% CI: 1.19-5.45) in multivariate analysis. For those individuals with euthyroidism, TPOAb was also associated with diabetes (OR=1.56, 95% CI: 1.06-2.29). What’s more, we demonstrated that TPOAb was associated with a lower prevalence of chronic lung disease and cancer in males (Chronic lung disease: OR=0.56, 95% CI: 0.34-0.93; cancer: OR=0.31, 0.15-0.65) and individuals older than 50 years old (Chronic lung disease: OR=0.44, 95% CI: 0.28-0.67; cancer: OR=0.54, 0.34-0.84). Consistent with the general population, TPOAb showed no association with hypertension, cardiovascular disease, arthritis and CKD stage 3-5 in subgroup analysis.

### Thyroid autoimmunity and mortality

The median duration of follow-up was 133 months. A total of 1121 all-cause deaths were observed, comprising 267 heart disease deaths, 279 cancer deaths, and 575 deaths from other causes. **Table 3** lists the relationship between thyroid autoantibodies and the risk of all-cause mortality in the Cox proportional regression models adjusted for covariates. In the univariate analysis, TgAb was associated with a higher risk of all-cause mortality (HR=1.75, 95% confidence interval [CI]: 1.29–2.38). However, this association disappeared after adjusting for confounders (HR=1.42, 95% CI: 0.96–2.10). By comparison, no statistically significant association was identified between TPOAb and all-cause mortality in the univariate and multivariate analysis (univariate: HR=1.09, 95% CI: 0.86–1.39; multivariate: HR=0.79, 95% CI: 0.59–1.04). We further analyzed the association between thyroid autoantibodies and heart disease mortality in **Table 4**. In the univariate analysis, thyroid TgAb was associated with increased heart disease mortality (HR=2.46, 95% CI: 1.40–4.33) while TPOAb did not exhibit significant association with heart disease mortality (HR=1.49, 95% CI: 0.94–2.35). However, after adjusting for confounders, both TgAb and TPOAb had no association with heart disease mortality (TgAb: HR=1.83, 95% CI: 0.93-3.61; TPOAb: HR=0.93, 95% CI: 0.56-1.55).

**Table 3:** Associations between different TGAb, TPOAb status and all-cause mortality in the National Health and Nutrition Examination Survey, 2007 to 2012 followed up through 2019.

Characteristics	Univariate		Multivariate*	
	HR	p	HR	p
**Age**	1.10 (1.09-1.11)	<0.01	1.09 (1.07-1.10)	<0.01
Gender				
Male	1	/	1	/
Female	0.68 (0.58-0.80)	<0.01	0.56 (0.47-0.66)	<0.01
Race				
Mexican American	1	/	1	/
Non-Hispanic White	3.28 (2.12-5.08)	<0.01	1.86 (1.27-2.72)	<0.01
Non-Hispanic Black	2.61 (1.70-4.00)	<0.01	1.72 (1.21-2.45)	<0.01
Other	1.29 (0.84-1.99)	0.24	1.07 (0.71-1.62)	0.74
Education status				
Under high school	1	/	1	/
High school or equivalent	0.57 (0.44-0.74)	<0.01	0.76 (0.59-0.98)	0.03
Above high school	0.41 (0.32-0.52)	<0.01	0.80 (0.60-1.06)	0.11
Poverty rartio				
**≤**1	1	/	1	/
1<to **≤** 3	1.54 (1.15-2.06)	<0.01	0.89 (0.69-1.14)	0.35
>3	0.73 (0.56-0.94)	0.02	0.59 (0.44-0.77)	<0.01
TSH				
0.34<to<5.6	1	/	1	/
≤0.34	2.34 (1.47-3.73)	<0.01	1.52 (0.96-2.41)	0.07
≥5.6	1.66 (1.04-2.66)	0.04	1.12 (0.64-1.94)	0.69
Smoking				
Never	1	/	1	/
Ever	2.55 (2.00-3.26)	<0.01	1.16 (0.91-1.47)	0.23
Current	1.62 (1.21-2.15)	<0.01	2.03 (1.58-2.61)	<0.01
BMI				
18.5≤BMI<25	1	/	1	/
BMI<18.5	2.59 (1.56-4.32)	<0.01	2.60 (1.46-4.64)	<0.01
BMI≥25	1.04 (0.82-1.31)	0.74	0.82 (0.63-1.07)	0.14
**Diabetes**	3.04 (2.53-3.66)	<0.01	1.33 (1.06-1.66)	0.01
**Hypertension**	3.69 (2.99-4.54)	<0.01	1.23 (0.98-1.53)	0.07
**Cardiovascular disease**	6.64 (5.28-8.37)	<0.01	1.76 (1.42-2.18)	<0.01
**Chronic lung disease**	1.51 (1.21-1.89)	<0.01	1.25 (1.02-1.54)	0.03
**Arthritis**	3.61 (2.97-4.40)	<0.01	1.22 (1.03-1.44)	0.02
**Cancer**	3.37 (2.72-4.17)	<0.01	1.03 (0.81-1.32)	0.80
**eGFR<60**	6.64 (5.39-8.16)	<0.01	1.20 (0.97-1.48)	0.09
**TGAb**	1.75 (1.29-2.38)	<0.01	1.42 (0.96-2.10)	0.08
**TPOAb**	1.09 (0.86-1.39)	0.73	0.79 (0.59-1.04)	0.09

*Multivariate analysis adjusted for age, sex, race, poverty index, education, smoking, BMI, diabetes, hypertension, CVD, chronic lung disease, arthritis, cancer, TSH, eGFR, TgAb and TPOAb.

**Table 4:** Associations between different TGAb, TPOAb status and heart disease mortality in the National Health and Nutrition Examination Survey, 2007 to 2012 followed up through 2019.

Characteristics	Univariate		Multivariate	
	HR	p	HR	p
**Age**	1.13 (1.11-1.15)	<0.01	1.11 (1.08-1.13)	<0.01
Gender				
Male	1	/	1	/
Female	0.71 (0.55-0.92)	0.01	0.46 (0.32-0.66)	<0.01
Race				
Mexican American	1	/	1	/
Non-Hispanic White	2.82 (1.34-5.93)	<0.01	1.40 (0.67-2.96)	0.36
Non-Hispanic Black	2.57 (1.23-5.37)	0.01	1.52 (0.74-3.12)	0.25
Other	1.11 (0.45-2.75)	0.82	0.90 (0.37-2.19)	0.81
Education status				
Under high school	1	/	1	/
High school or **eq**uivalent	0.71 (0.46-1.08)	0.11	1.03 (0.64-1.66)	0.90
Above high school	0.27 (0.20-0.38)	<0.01	0.59 (0.42-0.82)	<0.01
Poverty rartio				
**≤**1	1	/	1	/
1<to **≤** 3	1.87 (1.22-2.87)	<0.01	1.10 (0.70-1.71)	0.68
>3	0.63 (0.38-1.03)	0.07	0.65 (0.38-1.12)	0.12
**TSH**				
0.34<to<5.6	1	/	1	/
≤0.34	2.39 (0.89-6.38)	0.08	1.37 (0.47-4.01)	0.56
≥5.6	1.93 (0.73-5.11)	0.18	1.01 (0.37-2.76)	0.98
Smoking				
Never	1	/	1	/
Ever	2.08 (1.54-2.79)	<0.01	0.90 (0.66-1.23)	0.51
Current	1.23 (0.75-2.01)	0.41	1.91 (1.22-2.98)	0.01
BMI				
18.5≤BMI<25	1	/	1	/
BMI<18.5	0.35 (0.07-1.65)	0.18	0.37 (0.08-1.75)	0.20
BMI≥25	1.18 (0.77-1.81)	0.44	0.87 (0.57-1.35)	0.54
**Diabetes**	4.78 (3.14-7.28)	<0.01	1.81 (1.20-2.73)	<0.01
**Hypertension**	5.33 (3.69-7.70)	<0.01	1.34 (0.85-2.11)	0.20
**Cardiovascular disease**	11.13 (7.70-16.08)	<0.01	2.40 (1.63-3.54)	<0.01
**Chronic lung disease**	1.57 (1.00-2.45)	0.05	1.30 (0.81-2.07)	0.27
**Arthritis**	4.86 (3.46-6.84)	<0.01	1.41 (0.93-2.15)	0.11
**Cancer**	2.12 (1.36-3.32)	<0.01	0.57 (0.32-1.01)	0.06
**eGFR<60**	11.21 (7.87-15.96)	<0.01	1.53 (1.06-2.20)	<0.01
**TGAb**	2.46 (1.40-4.33)	<0.01	1.92 (1.04-3.56)	0.08
**TPOAb**	1.49 (0.94-2.35)	0.09	0.93 (0.56-1.52)	0.75

*Multivariate analysis adjusted for age, sex, race, poverty index, education, smoking, BMI, diabetes, hypertension, CVD, chronic lung disease, arthritis, cancer, TSH, eGFR, TgAb and TPOAb.

## Discussion

The results of this study suggest that thyroid autoantibodies are not only associated with thyroid disorders but they are also associated with extra-thyroid diseases. The results suggest that TgAb is associated with a higher prevalence of diabetes. TPOAb is also significantly associated with diabetes in certain subgroups (age <50 years old, euthyroid). Furthermore, TgAb has an inverse association with hypertension in multivariate analysis. TPOAb has an inverse association with chronic lung disease. In male individuals, both TgAb and TPOAb are associated with a lower prevalence of cancer. But both TgAb and TPOAb had no association with cardiovascular disease, arthritis and CKD stage 3-5. In univariate analysis, TgAb was associated with a higher risk of all-cause mortality. But this association disappears after adjusting for confounders. TPOAb showed no association with all-cause mortality in both univariate and multivariate analysis. In addition, both TgAb and TPOAb had no association with heart disease-specific mortality.

Some previous studies have also detected the association between thyroid diseases and diabetes. In a meta-analysis, the authors included 2972 young people and 789 adults with type 1 diabetes from 14 studies (28). They demonstrated a markedly increased risk of thyroid autoimmunity in people with type 1 diabetes. Another study also found a high prevalence of thyroid autoimmunity in type 1 diabetes (29). Type 1 diabetes is also a chronic autoimmune disease. The relationship between type 1 diabetes and thyroid autoimmunity may be largely explained by sharing a similar genetic inheritance (22). Studies to investigate a link between thyroid autoimmunity and type 2 diabetes have produced mixed results. A significantly higher prevalence of both TgAb and TPOAb was identified in a study of Saudi type 2 diabetes subjects by Whiles Akbar et al (30). One study demonstrated a high prevalence of TPOAb but not TgAb in Ghanaian type 2 diabetes patients compared to the general population (31). Another also demonstrated a positive association between TPOAb and type 2 diabetes (32). However, the study by Maryam Zahedi et al. did not yield a positive association between TPOAb and type 2 diabetes (33). In our study, we demonstrated a significant correlation between TgAb and diabetes while TPOAb only associated with diabetes diagnosed before 30 years old most of which was type 1 diabetes.

Few studies have explored the association between thyroid autoimmunity and hypertension. Most of the current studies focused on the association between thyroid function and hypertension. Thyroid dysfunction, including both hypo- and hyperthyroidism, may increase the risk of hypertension (34). We demonstrated that TgAb was inversely associated with the prevalence of hypertension. Maryam Tohidi et al. reported that elevated TPOAb levels can contribute to the development of hypertension among euthyroid men which was not demonstrated in our study (35). Another study by Yan Han et al. examined associations of maternal thyroid autoantibody positivity in the first and second trimesters with the risk of hypertensive disorder of pregnancy (36). They showed that TPOAb positivity and TgAb positivity in the first trimester are associated with an increased risk of hypertensive disorder of pregnancy. The exact mechanism by which thyroid autoantibodies affect the development of hypertension remains unknown. Genetic background may be a potential explanation. And antihypertensive drugs may also affect the thyroid autoantibodies. Further studies are required to clarify this issue.

Few studies have reported the association between thyroid autoimmunity and chronic lung disease. We demonstrated an inverse association between TPOAb and chronic lung disease. The exact mechanism is still unclear. Glucocorticoids have been widely used in the therapy of chronic lung disease due to their significant therapeutic effects (37). Long-term usage of glucocorticoids can cause immunosuppression which might play a role in reducing the development of autoimmune thyroid diseases (38). Whether other factors participate in the association between TPOAb and chronic lung disease still need further study.

We demonstrated that both TgAb and TPOAb were inversely associated with cancer in males. But this association was not observed in females. This divergence may be due to the spectrum and treatment difference between males and females. It is reported that thyroid autoimmunity was significantly associated with the risk of breast cancer (39). There was limited evidence on the association between thyroid autoimmunity and non-thyroid cancer in males. The treatment of cancer may contribute to the decreased prevalence of thyroid autoimmunity. It was reported that androgen deprivation therapy for prostate cancer was associated with a decreased risk of autoimmune diseases (40). However, the exact mechanism still needs further study.

In univariate analysis, we demonstrated that TgAb was an independent risk factor for all-cause mortality, while TPOAb did not show such an association. TgAb may affect survival by thyroid dysfunction. TgAb was associated with HT and GD. Hypothyroidism and thyrotoxicosis are the clinical hallmarks of HT and GD, respectively. Previous studies have reported that both hypothyroidism and hyperthyroidism are associated with a higher risk of mortality (41, 42). Individuals with thyroid autoimmunity were much older than those without thyroid autoimmunity, which can also affect survival. Furthermore, other risk factors for thyroid autoimmunity, such as excess iodine intake, radiation exposure, and others, can also be potential risk factors for mortality (43). Thyroid autoimmunity may also affect survival indirectly through these factors. After adjusting for confounders, neither TgAb nor TPOAb were found to be independent risk factors for all-cause mortality.

The major strengths of this study lie in the wealth of demographic and thyroid profiles in the large representative sample from the US population with a median 88-month follow-up. This is the first study to investigate the impact of thyroid autoimmunity on the risk of mortality with consideration of a multitude of potential confounding factors. However, there are several limitations to this study. First, thyroid antibodies vary over time. Baseline thyroid autoantibody status may not represent long-term average exposure, which can lead to misclassification of the exposure. In addition, we could not further analyze the association between the duration of thyroid autoantibodies and mortality. Second, the data on TSH receptor antibodies were not available in the NHANES, and we could not assess the impact of HT and GD on the risk of mortality. Third, the median follow-up time in the present study was only 88 months. The effect of thyroid autoimmunity on death should consider long-term effects, especially the specific cause of mortality. Fourth, the definition of extra-thyroid diseases (diabetes, hypertension, etc.), except for CKD, was simply based on responses to a standardized questionnaire in the NHANES database, rather than on clinical or pathological criteria. The reliance on self-reported data may not accurately embody the true prevalence or absence of these conditions, which could potentially affect the precision of the associations we observed. Fifth, the specific causes of death in the NHANES were defined using death certificates that were not confirmed by autopsy, which can result in misclassifications. To address these limitations, future research should aim for larger sample sizes, longer follow-up periods, and more robust criteria for defining diseases and causes of death. Such investigations would provide a more comprehensive understanding of the impact of thyroid autoimmunity on extra-thyroid diseases and mortality.

## Conclusion

In conclusion, using a nationally representative database of US adults, we found that positive TgAb was associated with a higher prevalence of diabetes and a lower prevalence of hypertension. TPOAb was associated with a lower prevalence of chronic lung disease. Neither TgAb nor TPOAb was a risk factor for all-cause mortality or heart disease mortality. Future studies are required to ascertain the mechanisms underlying the association between thyroid autoimmunity and extra-thyroid diseases.

## Data availability statement

Publicly available datasets were analyzed in this study. This data can be found here: https://www.cdc.gov/nchs/nhanes/about_nhanes.htm.

## Author contributions

J-LS: Data curation, Writing – original draft, Writing – review & editing. J-WH: Writing – original draft, Writing – review & editing, Data curation. L-RL: Formal analysis, Writing – review & editing. Z-LX: Data curation, Writing – review & editing. J-JL: Data curation, Writing – review & editing. S-RS: Investigation, Methodology, Project administration, Supervision, Writing – review & editing. CC: Data curation, Investigation, Methodology, Project administration, Supervision, Writing – review & editing.
