# Supplementary material for: Association of thyroid autoimmunity with extra-thyroid diseases and the risk of mortality among adults: evidence from the NHANES
Source: Front Endocrinol (Lausanne). 2024 Feb 9;15:1323994. doi: 10.3389/fendo.2024.1323994 (PMC10884096; doi:10.3389/fendo.2024.1323994)
Supplement: Supplementary file 1 [file Table_1.docx]

Supplement table 1 The association between thyroid autoimmunity and diabetes diagnosed before 30 and after 30.

|  | Univariate model | | Multivariate model | | | |
| --- | --- | --- | --- | --- | --- | --- |
|  |  |  | Model 1 | | Model 2 | |
|  | OR (95% CI) | p | HR (95% CI) | p | HR (95% CI) | p |
| Diabetes diagnosed before 30 |  |  |  |  |  |  |
| TgAb | 1.95 (0.77-4.94) | 0.15 | 2.28 (0.95-5.49) | 0.07 | 2.87 (1.12-7.33) | 0.03 |
| TPOAb | 2.26 (0.91-5.58) | 0.08 | 2.53 (1.03-6.22) | 0.04 | 3.01 (1.18-7.66) | 0.02 |
| Diabetes diagnosed after 30 |  |  |  |  |  |  |
| TgAb | 1.68 (1.11-2.56) | 0.02 | 1.54 (0.95-2.48) | 0.08 | 1.72 (1.03-2.88) | 0.04 |
| TPOAb | 1.14 (0.78-1.67) | 0.49 | 1.08 (0.72-1.64) | 0.70 | 1.11 (0.74-1.67) | 0.61 |
